# Improving somatic variant identification through integration of genome and exome data

**DOI:** 10.1186/s12864-017-4134-3

**Published:** 2017-10-16

**Authors:** Vinaya Vijayan, Siu-Ming Yiu, Liqing Zhang

**Affiliations:** 1 Genetics, Bioinformatics and Computational Biology, Virginia Tech, Blacksburg, VA USA; 2 Department of Computer Science, The University of Hong Kong, Pokfulam, Hong Kong; 3 Department of Computer Science, Virginia Tech, Blacksburg, VA USA

**Keywords:** Somatic variants, Genome and exome analysis, Framework for combining results from tools

## Abstract

**Background:**

Cost-effective high-throughput sequencing technologies, together with efficient mapping and variant calling tools, have made it possible to identify somatic variants for cancer study. However, integrating somatic variants from whole exome and whole genome studies poses a challenge to researchers as the variants identified by whole genome analysis may not be identified by whole exome analysis and vice versa. Simply taking the union or intersection of the results may lead to too many false positives or too many false negatives.

**Results:**

To tackle this problem, we use machine learning models to integrate whole exome and whole genome calling results from two representative tools, VCMM (with the highest sensitivity but very low precision) and MuTect (with the highest precision). The evaluation results, based on both simulated and real data, show that our framework improves somatic variant calling, and is more accurate in identifying somatic variants than either individual method used alone or using variants identified from only whole genome data or only whole exome data.

**Conclusion:**

Using machine learning approach to combine results from multiple calling methods on multiple data platforms (e.g., genome and exome) enables more accurate identification of somatic variants.

## Background

Somatic variants, unlike germline variants, are novel mutations that occur within a cell population and are not inherited. Identification of somatic variants enables the identification of variant hotspots. These hotspots can be used to study significant genes and pathways that can then be used in predictive, prognostic, remission and metastatic analysis of cancer. These somatic variant hotspots can also be used as therapeutic targets. Identifying somatic variants is more difficult than identifying germline variants because of copy number aberrations and the variability of somatic mutations.

In the past few years, a lot of methods have been developed to identify somatic variants. These programs differ in the kinds of statistics used and the parameters considered. For instance, SomaticSniper [1] uses a Bayesian approach to identify somatic variants. VarScan2 [2] uses Fisher’s test to differentiate germline variants from somatic variants and variants that lose heterozygosity. MuTect [3] predicts somatic variants by two Bayesian classifiers, taking into account that the variants are true mutations from the reference sequence and also are not present in normal samples. VCMM [4] uses a simple multinomial model to compare the probability of the variant being real variant with the probability of being sequencing error. Previous studies [5] have shown that MuTect is extremely conservative in its approach to identify somatic variants and has a high precision at the cost of identifying real somatic variants as germline. VCMM, on the other hand, tends to be liberal in its approach since it does not take into consideration the corresponding normal sample and hence identifies a lot of germline variants as somatic. Overall VCMM has the highest sensitivity while MuTect has the highest precision for detecting somatic variants [5–7].

The recent ICGC-TCGA Dream Mutation Calling challenge used crowd-sourcing to improve identification of somatic variants on one platform [8]. Different participating groups for the challenge have shown that ensemble approaches that integrate calling results from multiple somatic variant callers improve the identification of somatic variants over individual callers. For example, Kim et al. developed a statistical model that combines multiple callers [9]. SomaticSeq [10] uses Adaptive Boosting model and CAKE [11] uses majority voting to classify a variant as somatic. It is worth noting is that the aforementioned ensemble approaches have all been developed for identifying somatic variants from a single platform (i.e., a single data type, e.g., whole exome).

The low cost and high sequencing coverage associated with exome sequencing platform when compared to the whole genome sequencing platform, has lead to more exome sequencing than genome sequencing. However, a recent study has shown that the whole genome sequencing accurately identifies more germline variants than whole exome sequencing in the exon regions [12]. Even though the study is for germline variant identification, it nevertheless suggests that relying only on exome data for variant calling may miss many real variants. In fact, our previous study [5] has shown that the concordance between somatic variants identified by whole exome data and by exonic regions of whole genome data is at most only 11%, and almost 90% of the somatic variants are called by only one of these two platforms, suggesting that there is much room to explore with the two types of data for somatic variant calling. This phenomenon is also seen in germline variants, where concordance between whole exome and exonic regions of whole genome is low, ~53% [13]. The important question in these cases is which of the platform analysis should be trusted or rather how can we make the best use of the two types of data for better somatic variant calling whenever both data types are available?

To address this question, we develop a framework that integrates the whole exome data and the whole genome data for somatic variant calling. Using two commonly used somatic variant callers, MuTect and VCMM, the former shown to have the highest precision and the latter the highest sensitivity [5] to call somatic variants on both exome and genome data, we then extracted 108 features from the calling outputs of the two programs and used them as input to the machine learning algorithm to identify somatic variants.

## Results

Somatic mutations were generated on chromosome 1 of individual “A0BW” from TCGA [14]. The whole genome had 30X coverage and the whole exome had 150X coverage. Somatic mutations were generated using BAMSurgeon [8] at different depths and different allele fractions on whole genome and whole exome platforms (see Methods for details).

### Number of somatic variants identified by callers individually

The somatic mutations that are generated by BAMSurgeon and are also called by the somatic variant caller are considered as true positives. The false negatives (FN) are the simulated variants that were not called by the variant caller. The false positives (FP) are the variants called by the variant caller but are not simulated variants. All the sites that are not simulated variants and are not called by the variant caller are true negatives (TN). Sensitivity was calculated using the formula TP/(TP + FN). Precision was calculated using the formula TP/(TP + FP). F1-score is calculated using the formula (2*precision*recall)/(precision + recall). Table 1 shows the number of somatic variants identified by different somatic variant callers for the simulated whole genome and whole exome samples. Out of all the methods, VCMM has the highest sensitivity of 0.78 but very low precision while MuTect has the highest precision value of 0.88 (similar to SomaticSniper). To improve the effectiveness of our framework, we decided to combine the results from MuTect and those from VCMM.

**Table 1:** The number of somatic variants called by four methods

Method	# of True positives^a^	# of Somatic Variants^b^	Sensitivity	Precision
MuTect	538	614	0.77	0.88
SomaticSniper	540	617	0.77	0.88
VarScan2	527	603	0.75	0.87
VCMM	548	399,491	0.78	0.0014

^a^Number of true positives, computed as the simple union of the number of variants called by each method out of the 700 simulated somatic variants for the two platform data

^b^Total number of somatic variants called by each method on the whole exome and whole genome datasets

### Results from different machine learning models

**Fig. 1:**
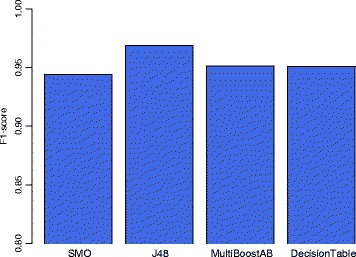
F1-score for different machine learning models

Figure 1 shows the results of a 10-fold cross-validation procedure using different machine learning classification models to identify true somatic variants. All classification algorithms used for this study were implemented in Waikato Environment for Knowledge Analysis (WEKA). There were altogether 570,575 positions that were considered by MuTect and VCMM for somatic variant calling. The training set was built by randomly selecting 10,000 positions from the 570,575 positions. MuTect and VCMM were applied to the 10,000 sites. From the results of these two callers, 108 features were collected and used for machine learning. Comparison of different classifiers (only results of SMO, J48, MultiBoostAB, and DecisionTable with F1-scores higher than 0.90 are shown for brevity) shows that J48 has the highest F1-score of 0.968, and therefore was chosen as the classifier in the current study for further analysis.

### Reason for integration of multiple tools and multiple datasets

**Fig. 2:**
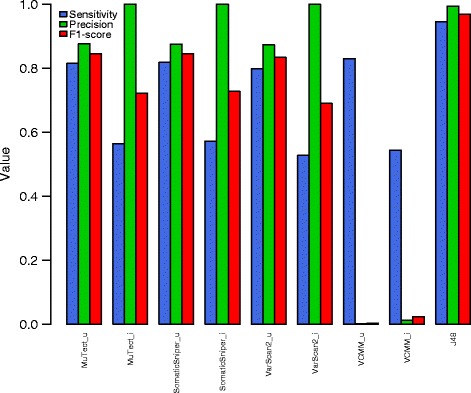
Sensitivity, precision and F1-score with MuTect, SomaticSniper, VarScan2 VCMM and J48. ‘u’ indicates union and ‘i’ indicates intersection results

**Fig. 3:**
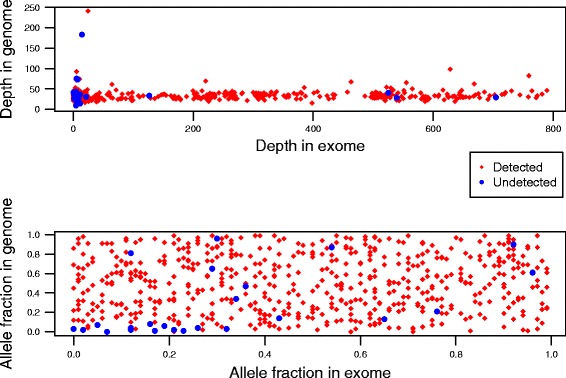
Distribution of the true positives and false negatives across the depth and allele fractions of whole genome and whole exome. Red rhombus depict true positives and blue dots depict false negatives

Figure 2 shows the results of using J48 when compared to simple union and simple intersection of variants identified from only MuTect, SomaticSniper, VarScan2, and VCMM. Using J48 gives a sensitivity, precision, and F1-score of 0.94, 0.99 and 0.968, respectively. A union of somatic variants using MuTect, SomaticSniper, VarScan2, and VCMM gives an F1-score of 0.84, 0.84, 0.83, and 0.002 respectively; and 0.72, 0.72, 0.69 and 0.02 respectively for simple intersection. This shows that our ensemble method which integrates multiple tools is better than individual callers in both sensitivity and precision. Figure 3 shows a distribution of the simulated variants that were detected by J48 (i.e., true positives) and the simulated variants that were missed by J48 (i.e., false negatives) across the coverage depth and allele fractions of the whole genome and the whole exome. Most somatic variants that J48 could not call as somatic had a low allele fraction in the genome and a low exome depth.

**Fig. 4:**
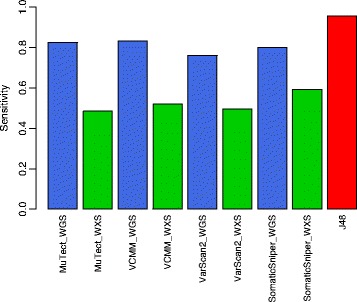
Performance comparison of somatic variant identification for single platform, i.e., whole genome (WGS) or whole exome (WXS) versus ensemble method

Figure 4 shows why combining whole genome and whole exome variants is better than calling variants from only one platform. Only somatic variants that were simulated on both whole genome and whole exome were considered for this part of the analysis. As seen in Fig. 4, if only whole exome was considered the maximum sensitivity obtained was 0.59 (SomaticSniper) while if only whole genome was considered the maximum sensitivity obtained was 0.83 (VCMM). Using simple union of both whole genome and whole exome variant calling gives a highest sensitivity of 0.93 (VCMM). Using J48 to integrate somatic variants from both whole genome and whole exome platforms helps achieve a sensitivity of 0.95. This clearly shows that integrating datasets from multiple platforms is better than just considering variants from one particular platform.

### Results for cross-contamination of normal samples

It is known that Mutect imposes a heavy penalty on variants that are also present in the normal samples to prevent cross-contamination. However, this practice can also miss the true somatic variants and thus increase the number of false negatives. Therefore, it is necessary to study the effect of cross contamination on variant calling for the integrated caller. The normal samples were hence contaminated with allelic reads from the tumor samples at different percentages i.e. 2.5%, 5%, 7.5%, and 10%. It is observed that as the degree of contamination increases, the F1-score reduces. Normal samples whose reads were replaced with allelic reads from the tumor samples at 2.5%, 5%, 7.5%, and 10% had 10-fold cross-validation scores of F1-scores of 0.96, 0.95, 0.93, 0.91 (Sensitivity- 0.93, 0.92, 0.89, 0.86 and Precision- 0.99, 0.98, 0.97, 0.97) respectively.

### Comparison with similar tools

**Fig. 5:**
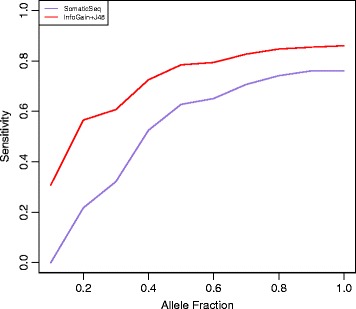
Performance comparison of SomaticSeq versus our ensemble method on only whole genome platform

Since our framework is the first of its kind in integrating both multiple tools and multiple platforms, we could not compare our framework to another software in the same domain. We compared J48 to SomaticSeq [10], a tool that uses machine learning (Adaptive Boosting model implemented in R) to integrate somatic variant calling from multiple tools (MuTect, JointSNVMix2, SomaticSniper, VarDict, and VarScan2) from only whole genome or only whole exome platform to identify somatic variants. We applied SomaticSeq using the default trained model built from high quality synthetic data. To make a fair comparison, we only compared somatic variants simulated in the non-exonic regions of the genome. Figure 5 shows that J48 performs better than SomaticSeq, achieving a sensitivity of 0.86 against SomaticSeq’s 0.76.

### Real data validation

For real data, we do not know the true somatic variants. Hence, variants that were identified by at least two somatic variant callers out of four somatic variant callers i.e. MuTect, SomaticSniper, VarScan2, and VCMM and from at least two platforms out of the three platforms (i.e., WGS, WXS and validation BAM files) available on TCGA were treated as true somatic variants (positives). Variants that were identified by only one platform or by only one somatic variant caller but were covered by another platform by a depth of at least 10X were considered to be true negatives. Our ensemble method based on a 10-fold cross validation on real data gives sensitivity, precision and F1-score of 0.85, 0.80, and 0.83 respectively for A15K.

One of the ways to verify that the ensemble method works better than using only individual somatic variant caller is to show that the ensemble method can find somatic point mutations present in the whole genome but not in the whole exome despite these regions being covered by the whole exome sequencing. To make sure that the regions were true somatic point mutations, we searched for regions that were called by more than two somatic variant callers (MuTect, SomaticSniper, VarScan2, and VCMM) for the whole genome and also were present in the COSMIC database. We found 50 such positions that were identified by more than two somatic variant callers in the whole genome of individual “A15K” and were present in the COSMIC database but were not called by any of the somatic variant callers from the whole exome BAM file even though they were covered by the whole exome sequencing. This shows our ensemble approach identifies variants that would have been missed if an intersection of the whole genome and whole exome somatic variants was considered as the method for the identification of somatic point mutations.

### Robustness of the ensemble method

**Fig. 6:**
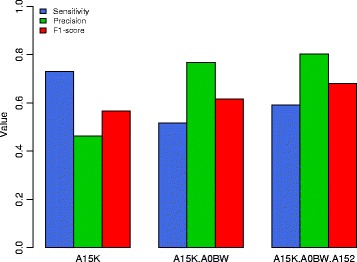
Sensitivity, precision and F1-scores based on different training sets: (i) A15K, (ii) combination of A15K-A0BW, and (iii) combination of A15K-A0BW-A152, using A15E as the test set

We also assessed the performance of our ensemble method using the same training set mentioned above but another test dataset to examine the robustness of the trained model. To do this we produced a test dataset from another individual (A15E) from TCGA. The test dataset was produced using the MuTect, SomaticSniper, VarScan2 and VCMM results from individual A15E using the procedure mentioned above, i.e., variants from at least 2 callers on at least 2 platforms are positives while variants identified on only one platform by only one somatic variant caller and covered by a depth of at least 10X are negatives. We used the training set combined from A15K, and the datasets from A15E as test sets to check for the performance using the training set. This gave an F1-score of 0.567 (Fig. 6). To increase the robustness of the training set, the AOBW dataset was added to A15K. This was tested on A15E, which gives an F1-score of 0.617. We then added A152 dataset to the combination of A15K and A0BW. The combination of A15K-A0BW-A152 was tested on A15E, which gives an F1-score of 0.681 with a precision of 0.803 and a sensitivity 0.591. Additional file 1: Table S1 and Additional file 2: Figure S1 show that J48 has better precision than Varscan2, VCMM, and SomaticSniper. Even though MuTect has a higher precision, MuTect identifies only 70 somatic variants as compared to 220 by J48. J48 has higher sensitivity than MuTect, and Varscan2. SomaticSniper and VCMM have a higher sensitivity than J48 at the expense of identifying more false positives than J48.

## Discussion

In somatic variant calling, an ideal case scenario would be if a variant is identified on both whole genome and whole exome platforms as this would also be a strong corroboration of a somatic variant at that particular position. However, in reality, there could be variants that are identified in the whole genome data but not identified in the whole exome and vice-versa. In fact, it has been shown that germline variants called on both platforms account for only 53% of the total variants called on the two platforms [13], and for somatic variants, the proportion of variants that are called on both platforms is even smaller, only about 11% [5]. One of the reasons for the low consensus could be the region not being sequenced at a high enough coverage in either of the platforms. If the allele fraction is too low, the allele may not have enough coverage in one of the platforms and thus may have been considered as a sequencing error. The region can be a low complexity region such as a repeat region and hence, is difficult to identify. To address this issue and overcome the disagreement between different platforms, we present a framework that can be used to facilitate our aim to improve the identification of somatic variants.

Generally, either an intersection or union of called variants from whole genome and whole exome platforms is taken into account to identify variants for an individual. However, using a simple union of variants could lead to the incorporation of many false positives; using simple intersection could lead to the exclusion of many true positives, especially considering the observation that the concordance between exome and genome within the exonic regions did not exceed 11% for any of the commonly used somatic variant callers [5]. It has been shown that more variants are identified from the exonic regions of the whole genome than the whole exome in the case of germline variants [15]. Our study corroborates this in case of somatic variants (Fig. 4). Integration of multiple variant calling tools is better than using a tool individually has also been shown before [11]. Hence, we provide a framework that uses an ensemble approach to incorporate variants from both whole exome and whole genome platforms using multiple somatic variant callers without adding too many false positives and missing too many true positives.

To resolve the disagreement between the variants detected between the two platforms, we developed an ensemble method that combines the outputs from MuTect and VCMM. The output file of MuTect using the “call_stats” options gives details of the variants in normal and tumor samples‚ and reasons for why a variant was accepted as a somatic variant or why it was rejected. We focus on integrating MuTect and VCMM since it has been shown that MuTect has a high precision while VCMM has a high sensitivity [5]. VCMM predicts 100 times the number of somatic variants that MuTect, VarScan2 or SomaticSniper predict and thus, predicts a lot of false positives. VCMM does not take the normal sample into consideration and hence predicts a lot of somatic variants. According to our previous study around 80% of the variants that VCMM predicts are germline variants. So it is important to restrict the number of somatic variants that VCMM predicts with the help of MuTect, which heavily penalizes the presence of variants in normal samples. On the other hand, since MuTect is extremely conservative in its approach, it is necessary to use VCMM to increase the identification of true somatic variants.

We used J48 in our ensemble approach to classify the variants identified by MuTect and VCMM as somatic or not. Decision tree J48 in WEKA is an iterative Dichotomiser 3 (ID3) implementation. Decision trees are very advantageous since they can handle missing values and many types of data including nominal, numeric, and textual data. Our input data to J48 was obtained from the output of MuTect and VCMM on the whole genome and whole exome data. The input data to J48 includes a lot of numeric and textual data. Attributes such as base quality and mapping quality are numerical while attributes such as dbSNP and COSMIC are textual. If a variant was identified by MuTect but not identified by VCMM or vice-versa, or if it is identified on only one of the platforms out of whole genome and whole exome, the attributes could have a lot of missing data. A decision tree uses information gain for attribute selection. Information gain assigns an importance to each attribute by giving a cutoff value to each attribute to split the node into two leaves. We used all the 108 features, i.e., all the information collected from MuTect and VCMM output files. The 108 features include base quality, mapping quality, indel score, SNP quality, allele fraction, coverage of normal and tumor samples, presence of the position in dbSNP or COSMIC database, and so on. These features were selected because most somatic variant callers use different and arbitrary cutoffs for the features. With increased read length, the mapping quality would increase which would only help improve the accuracy of somatic variant calling by individual somatic variant callers. Since, the accuracy of our method is dependent on somatic variant calling by VCMM and MuTect, increased read lengths would help increase in somatic variant calling. The minimum coverage for the ensemble method to correctly identify somatic variants is dependent on the identification of somatic variants by MuTect and VCMM. The minimum depth and allelic depth required by VCMM is 5 and 2 respectively. The minimum allelic depth required by MuTect is 2. It should be noted that depth alone cannot help identification of a somatic variant correctly. Factors like allele fraction, quality score, strand bias, mapping quality among others will affect its identification. We let the machine learning algorithm to decide a cutoff and to determine whether the variants are truly somatic (Additional file 3: Document 1).

We show that the ensemble approach gives a high F1 score on both simulated and real data (Fig. 2 and Fig. 6). Using an ensemble approach reduces the number of false positives and hence the precision values of the ensemble method increases compared to precision values of individual callers (Fig. 2). The sensitivity values are limited by the callers that are used in the ensemble method because the variants that are not identified by any method individually cannot be identified by the ensemble approach either. Intuitively, it makes sense to predict that variants that would not be detected either had a low allelic fraction or a low tumor sequencing depth in the whole genome or whole exome platforms, which is seen in our study (Fig. 3). We also compared our framework to SomaticSeq, also a machine learning framework that combines results from multiple variant calling tools to identify variants from whole genome or whole exome. Our framework performs better than SomaticSeq to identify somatic variants on one platform (Fig. 5). This shows that our framework of combining Mutect and VCMM can also be used effectively to identify somatic variants accurately from only one platform. Although we demonstrated our framework using VCMM and MuTect, we believe that our framework can also be applied to other tools.

In a clinical set up, it is still uncommon to obtain both normal and tumor samples because of the costs associated with sequencing normal and tumor tissue samples. Identifying somatic variants from only tumor samples is difficult because it would be challenging to differentiate between germline variants, somatic variants, and sequencing errors. VCMM does not take into account the normal samples while identifying somatic variants. MuTect can also be used to identify somatic variants without the normal sample but this results in lower accuracy. A future direction for this work would be to improve identification of somatic variants using variants identified from only tumor samples.

## Conclusions

We developed a framework that integrates somatic point mutations called by two somatic variant callers MuTect and VCMM from two platforms i.e., whole genome and whole exome. We used 108 attributes from the MuTect and VCMM outputs as input to decision tree classifier J48 to classify the variants from MuTect and VCMM as truly somatic or not. This ensemble method works better than using individual calling methods, or using the simple union or intersection of variants called by the methods. Using this ensemble approach only on whole genome or only whole exome platforms also works better than using only one method individually, showing that the approach is promising.

## Methods

### Generating simulated dataset

**Fig. 7:**
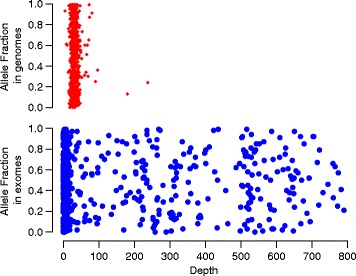
Distribution of simulated somatic point mutations across different allele fractions and depths on whole genome and whole exome platforms

Simulated datasets for the whole exome and whole genome platforms were generated using BAMSurgeon. BAMSurgeon can add somatic variants to genome and exome platforms by adding mutations to particular sites at specified allele fraction. Somatic variants were simulated on chromosome 1 of the individual “A0BW” from TCGA. Real exome data usually have different coverage depths depending on the particular experiments. Therefore, 100 somatic variants were generated with coverage depths <=8×, <=14×, <=200×, <=500×, <=800×, and >800× for the whole exome data. Therefore, to closely reflect the observation in real data, 100 variants were also simulated in regions that were covered by the whole genome but not by the whole exome. Since difference in coverage depth between normal and tumor samples is also common in real data, to closely mimic the real data, a coverage difference of a maximum of 50% was set between the normal and tumor samples. The parameter *coverdiff* in BAMSurgeon is utilized to simulate the coverage depth difference between tumor and normal samples. Note that the difference in coverage between normal and tumor, the difference in depth and allele fraction in whole exome and genome is a representation of real data. Figure 7 shows the distribution of simulated somatic point mutations across different allele fractions and coverage depths on whole genome and whole exome platforms.

### Building training and test sets for simulated data

The simulated variants are considered as true somatic variants (positives). Variants that were heterozygous in the normal sample or germline variants and other sites detected as variants by Mutect and VCMM were identified as not somatic (negatives). The germline variants in the sample were identified using GATK HaplotypeCaller since it has been shown to identify germline variants with high precision [16]. Output results from the “call_stats” option of Mutect and VCMM were then combined and the resulting file contains variants accepted as somatic variants by Mutect, variants rejected by Mutect due to various reasons, and variants called by VCMM. Altogether 108 features such as coverage depth, mapping quality, base quality, indel score, presence in dbSNP or COSMIC database, and number of bases in positive or negative strand were used to build the training set. The list of the 108 features as ranked by the InfoGain algorithm is shown in Additional file 1: Table S2. A custom code used to build this features list is available at https://bioinformatics.cs.vt.edu/zhanglab/software.html.

### Models used to identify somatic variants

A number of classification tools were tested to identify the most suitable model that can be used to identify somatic variants. The classification algorithms that were tested were “J48”, “SM0”, “DecisionTable” and “AdaBoostM1”. These algorithms were implemented as a part of the WEKA suite [17].

### Building training and test sets for real data

To examine the performance of the machine learning model, real data was also used. The DNA of the individual “A15K” have been sequenced three different times on different platforms, i.e., whole genome, whole exome, and validation BAM files (available on TCGA). Since we do not know the actual somatic variants in an individual with tumor, variants were considered as positives (i.e., truly somatic) if they were called as somatic by at least two methods (out of Mutect, SomaticSniper, Varscan2, and VCMM) on at least two platforms (whole genome, whole exome, and validation BAM files). Using variants from multiple platforms by multiple callers as true variants in real data has been used before by different studies [18]. Variants were considered as negatives (i.e., not somatic) if they were identified as somatic by only one method on one platform and had a coverage depth of at least 10X in the normal and tumor samples on the other two platforms. For example, if a variant was identified by only one caller on whole genome but was not identified by any other caller on whole exome or validation BAM files, the variant was considered as negative. This 10X coverage was selected because most methods require a minimum depth of 8-12X [19] to identify somatic variants.

Another test data was generated to validate the training set. The individual “A15E” was used for this purpose. The methodology described above to identify true and false somatic variants was used to build the test data, i.e., variants identified as somatic by multiple callers from multiple platforms are considered as positives while variants identified by only one somatic variant caller on one platform but not identified by any other variant caller on the other two platforms are considered as negatives.

To build a robust training set for real data, we combined variants from three real datasets i.e. A15K, A0BW and A152. 2419 number of true positive examples and 26,637 number of negative examples were included in the training set. We built another robust training set for real data, since it would be difficult to encompass the distribution in base quality, mapping quality, allele fraction, low coverage of alleles, nearby indels, nearby repeat regions in simulated data.

## Additional files

Additional file 1: Table S1. The number of true positives, false positives, false negatives identified by five methods for A15E dataset. A15K-A0BW-A152, was used as the training set for J48. **Table S2.** The number of features as ranked by InfoGain algorithm. *G indicates genome, X indicates exome, M indicates MuTect, V indicates VCMM. See [4, 20] for further deails on the parameters. (DOCX 508 kb)

Additional file 2: Figure S1. Sensitivity, precision and F1-scores using MuTect, SomaticSniper, VarScan2, VCMM, and J48 with A15E as the test set. A15K-A0BW-A152, was used as the training set for J48. (PDF 106 kb)

Additional file 3 Document 1. shows the tree built by J48 using the model training set. (DOCX 487 kb)

## Abbreviations

COSMIC: Catalogue Of Somatic Mutations In Cancer
FNs: False Negatives
FPs: False Positives
ID3: Iterative Dichotomiser 3
TCGA: The Cancer Genome Atlas
TNs: True Negatives
TPs: True Positives
VCMM: Variant Caller with Multinomial probabilistic Model
WEKA: Waikato Environment for Knowledge Analysis
WGS: Whole Genome Sequence
WXS: Whole Exome Sequence
