# Supplementary material for: Improving somatic variant identification through integration of genome and exome data
Source: BMC Genomics. 2017 Oct 16;18(Suppl 7):748. doi: 10.1186/s12864-017-4134-3 (PMC5657037; doi:10.1186/s12864-017-4134-3)
Supplement: Supplementary file 1 — The number of true positives, false positives, false negatives identified by five methods for A15E dataset. A15K-A0BW-A152, was used as the training set for J48. Table S2. The number of features as ranked by InfoGain algorithm. *G indicates genome, X indicates exome, M indicates MuTect, V indicates VCMM. See [4, 20] for further deails on the parameters. (DOCX 508 kb) [file 12864_2017_4134_MOESM1_ESM.docx]

**Additional files**

**Additional file 1:** Table S1: The number of true positives, false positives, false negatives identified by five methods for A15E dataset. A15K-A0BW-A152, was used as the training set for J48.

| **Method** | **True Positives** | **False Positives** | **False Negatives** | **Sensitivity** | **Precision** | **F1-score** |
| --- | --- | --- | --- | --- | --- | --- |
| MuTect | 77 | 11 | 294 | 0.20 | 0.875 | 0.33 |
| SomaticSniper | 339 | 996 | 32 | 0.91 | 0.25 | 0.39 |
| VarScan2 | 206 | 126 | 165 | 0.55 | 0.62 | 0.58 |
| VCMM | 366 | 1565 | 5 | 0.98 | 0.18 | 0.31 |
| J48 | 220 | 54 | 154 | 0.58 | 0.80 | 0.67 |

**Additional file 1:** Table S2: The number of features as ranked by InfoGain algorithm. *G indicates genome, X indicates exome, M indicates MuTect, V indicates VCMM. See [4, 20] for further details on the parameters.

| Rank | Parameter | Description* |
| --- | --- | --- |
| 1 | tot_depthG | depth (G,V) |
| 2 | refG | Reference allele (G,V) |
| 3 | altG | Alternate allele(G,V) |
| 4 | p_allG | log10(p-allele)(G,V) |
| 5 | p_errG | log10(p-error) (G,V) |
| 6 | snp_qualG | SNP-quality (G,V) |
| 7 | ind_filterG | neighbor-indel-filter (G,V) |
| 8 | snp_filterG | neighbor-snp-filter (G,V) |
| 9 | tot_depthX | depth (X,V) |
| 10 | refX | Reference allele (X,V) |
| 11 | altX | Alternate allele(X,V) |
| 12 | p_allX | log10(p-allele)(X,V) |
| 13 | p_errX | log10(p-error) (X,V) |
| 14 | snp_qualX | SNP-quality (X,V) |
| 15 | ind_filterX | neighbor-indel-filter (X,V) |
| 16 | snp_filterX | neighbor-snp-filter (X,V) |
| 17 | scoreX | score (X,M) |
| 18 | dbsnp_siteX | dbsnp_site (X,M) |
| 19 | coveredX | covered (X,M) |
| 20 | powerX | power (X,M) |
| 21 | tumor_powerX | tumor_power (X,M) |
| 22 | normal_powerX | normal_power (X,M) |
| 23 | normal_power_nspX | normal_power_nsp (X,M) |
| 24 | normal_power_wspX | normal_power_wsp (X,M) |
| 25 | total_readsX | total_reads (X,M) |
| 26 | map_Q0_readsX | map_Q0_reads (X,M) |
| 27 | init_t_lodX | init_t_lod (X,M) |
| 28 | t_lod_fstarX | t_lod_fstar (X,M) |
| 29 | t_lod_fstar_forX | t_lod_fstar_for (X,M) |
| 30 | t_lod_fstar_revX | t_lod_fstar_rev (X,M) |
| 31 | tumor_fX | tumor_f (X,M) |
| 32 | contaminant_fracX | contaminant_frac (X,M) |
| 33 | contaminant_lodX | contaminant_lod (X,M) |
| 34 | t_q20_countX | t_q20_count (X,M) |
| 35 | t_ref_countX | t_ref_count (X,M) |
| 36 | t_alt_countX | t_alt_count (X,M) |
| 37 | t_ref_sumX | t_ref_sum (X,M) |
| 38 | t_alt_sumX | t_alt_sum (X,M) |
| 39 | t_ref_max_mapqX | t_ref_max_mapq (X,M) |
| 40 | t_alt_max_mapqX | t_alt_max_mapq (X,M) |
| 41 | t_ins_countX | t_ins_count (X,M) |
| 42 | t_del_countX | t_del_count (X,M) |
| 43 | normal_best_gtX | normal_best_gt (X,M) |
| 44 | init_n_lodX | init_n_lod (X,M) |
| 45 | normal_fX | normal_f (X,M) |
| 46 | n_q20_countX | n_q20_count (X,M) |
| 47 | n_ref_countX | n_ref_count (X,M) |
| 48 | n_alt_countX | n_alt_count (X,M) |
| 49 | n_ref_sumX | n_ref_sum (X,M) |
| 50 | n_alt_sumX | n_alt_sum (X,M) |
| 51 | Power_Pos_SBX | power_to_detect_positive_strand_artifact (X,M) |
| 52 | Power_Neg_SBX | power_to_detect_negative_strand_artifact (X,M) |
| 53 | SB1X | strand_bias_counts1 (X,M) |
| 54 | SB2X | strand_bias_counts2 (X,M) |
| 55 | SB3X | strand_bias_counts3 (X,M) |
| 56 | SB4X | strand_bias_counts4 (X,M) |
| 57 | AT_FmedX | tumor_alt_fpir_median (X,M) |
| 58 | AT_FmadX | tumor_alt_fpir_mad (X,M) |
| 59 | AT_RmedX | tumor_alt_rpir_median (X,M) |
| 60 | AT_RX | tumor_alt_rpir_mad (X,M) |
| 61 | NormX | observed_in_normals_count (G,M) |
| 62 | judgementX | judgement (G,M) |
| 63 | scoreG | score (G,M) |
| 64 | dbsnp_siteG | dbsnp_site (G,M) |
| 65 | coveredG | covered (G,M) |
| 66 | powerG | power (G,M) |
| 67 | tumor_powerG | tumor_power (G,M) |
| 68 | normal_powerG | normal_power (G,M) |
| 69 | normal_power_nspG | normal_power_nsp (G,M) |
| 70 | normal_power_wspG | normal_power_wsp (G,M) |
| 71 | total_readsG | total_reads (G,M) |
| 72 | map_Q0_readsG | map_Q0_reads (G,M) |
| 73 | init_t_lodG | init_t_lod (G,M) |
| 74 | t_lod_fstarG | t_lod_fstar (G,M) |
| 75 | t_lod_fstar_forG | t_lod_fstar_forward (G,M) |
| 76 | t_lod_fstar_revG | t_lod_fstar_reverse (G,M) |
| 77 | tumor_fG | tumor_f (G,M) |
| 78 | contaminant_fracG | contaminant_fraction (G,M) |
| 79 | contaminant_lodG | contaminant_lod (G,M) |
| 80 | t_q20_countG | t_q20_count (G,M) |
| 81 | t_ref_countG | t_ref_count (G,M) |
| 82 | t_alt_countG | t_alt_count (G,M) |
| 83 | t_ref_sumG | t_ref_sum (G,M) |
| 84 | t_alt_sumG | t_alt_sum (G,M) |
| 85 | t_ref_max_mapqG | t_ref_max_mapq (G,M) |
| 86 | t_alt_max_mapqG | t_alt_max_mapq (G,M) |
| 87 | t_ins_countG | t_ins_count (G,M) |
| 88 | t_del_countG | t_del_count (G,M) |
| 89 | normal_best_gtG | normal_best_gt (G,M) |
| 90 | init_n_lodG | init_n_lod (G,M) |
| 91 | normal_fG | normal_f (G,M) |
| 92 | n_q20_countG | n_q20_count (G,M) |
| 93 | n_ref_countG | n_ref_count (G,M) |
| 94 | n_alt_countG | n_alt_count (G,M) |
| 95 | n_ref_sumG | n_ref_sum (G,M) |
| 96 | n_alt_sumG | n_alt_sum (G,M) |
| 97 | Power_Pos_SBG | power_to_detect_positive_strand_artifact (G,M) |
| 98 | Power_Neg_SBG | power_to_detect_negative_strand_artifact (G,M) |
| 99 | SB1G | strand_bias_counts1 (G,M) |
| 100 | SB2G | strand_bias_counts2 (G,M) |
| 101 | SB3G | strand_bias_counts3 (G,M) |
| 102 | SB4G | strand_bias_counts4 (G,M) |
| 103 | AT_FmedG | tumor_alt_fpir_median (G,M) |
| 104 | AT_FmadG | tumor_alt_fpir_mad (G,M) |
| 105 | AT_RmedG | tumor_alt_rpir_median (G,M) |
| 106 | AT_RG | tumor_alt_rpir_mad (G,M) |
| 107 | NormG | observed_in_normals_count (G,M) |
| 108 | judgementG | judgement (G) |
