# Supplementary figures and images for: Improving somatic variant identification through integration of genome and exome data
Source: BMC Genomics. 2017 Oct 16;18(Suppl 7):748. doi: 10.1186/s12864-017-4134-3 (PMC5657037; doi:10.1186/s12864-017-4134-3)

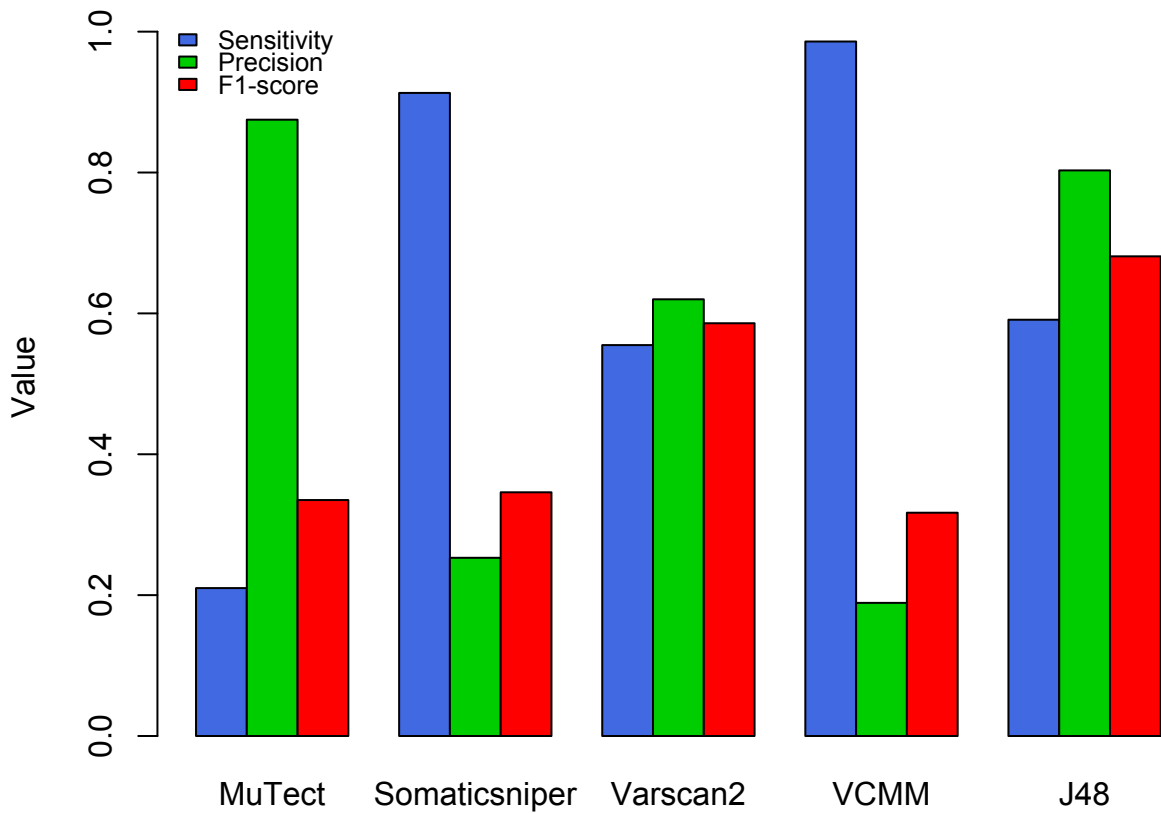

Supplement: Supplementary file 2 — Sensitivity, precision and F1-scores using MuTect, SomaticSniper, VarScan2, VCMM, and J48 with A15E as the test set. A15K-A0BW-A152, was used as the training set for J48. (PDF 106 kb) [file 12864_2017_4134_MOESM2_ESM.pdf]
