# Supplementary material for: Improving somatic variant identification through integration of genome and exome data
Source: BMC Genomics. 2017 Oct 16;18(Suppl 7):748. doi: 10.1186/s12864-017-4134-3 (PMC5657037; doi:10.1186/s12864-017-4134-3)
Supplement: Supplementary file 3 — Document 1. shows the tree built by J48 using the model training set. (DOCX 487 kb) [file 12864_2017_4134_MOESM3_ESM.docx]

The tree built by J48 using the model training set i.e. A0BW-A15K-A152

judgementG = KEEP

| init_t_lodG <= 5.089038

| | Power_Pos_SBG <= 0.801398: DISCARD (188.02/6.12)

| | Power_Pos_SBG > 0.801398

| | | AT_FmadG <= 15.5

| | | | coveredX = COVERED: DISCARD (16.72/3.01)

| | | | coveredX = UNCOVERED

| | | | | t_alt_sumG <= 195: DISCARD (2.25/0.0)

| | | | | t_alt_sumG > 195: ACCEPT (3.27/0.27)

| | | AT_FmadG > 15.5: ACCEPT (4.04/0.04)

| init_t_lodG > 5.089038

| | n_alt_sumX <= 26: ACCEPT (806.46/7.65)

| | n_alt_sumX > 26

| | | t_ref_max_mapqX <= 52: DISCARD (2.85/0.11)

| | | t_ref_max_mapqX > 52: ACCEPT (23.63/4.01)

judgementG = REJECT

| n_alt_sumG <= 202

| | total_readsG <= 1434

| | | normal_fG <= 0.218014

| | | | snp_qualG <= 11.373

| | | | | t_del_countG <= 5

| | | | | | tumor_fG <= 0.16129

| | | | | | | snp_qualG <= 7.732: DISCARD (619.02/47.59)

| | | | | | | snp_qualG > 7.732

| | | | | | | | tumor_fX <= 0.122222

| | | | | | | | | map_Q0_readsG <= 9

| | | | | | | | | | normal_power_nspG <= 0.99649

| | | | | | | | | | | t_ref_max_mapqG <= 48: DISCARD (2.48/0.36)

| | | | | | | | | | | t_ref_max_mapqG > 48: ACCEPT (24.67/9.58)

| | | | | | | | | | normal_power_nspG > 0.99649

| | | | | | | | | | | coveredX = COVERED: DISCARD (179.59/33.6)

| | | | | | | | | | | coveredX = UNCOVERED

| | | | | | | | | | | | normal_power_nspX <= 0: ACCEPT (7.32/1.15)

| | | | | | | | | | | | normal_power_nspX > 0: DISCARD (8.67/1.76)

| | | | | | | | | map_Q0_readsG > 9: DISCARD (35.59/0.18)

| | | | | | | | tumor_fX > 0.122222

| | | | | | | | | SB4G <= 0: DISCARD (15.87/7.23)

| | | | | | | | | SB4G > 0: ACCEPT (20.25/2.7)

| | | | | | tumor_fG > 0.16129

| | | | | | | total_readsX <= 35

| | | | | | | | powerX <= 0.099721

| | | | | | | | | dbsnp_siteG = NOVEL

| | | | | | | | | | snp_qualG <= 6.963: DISCARD (2.53/0.18)

| | | | | | | | | | snp_qualG > 6.963: ACCEPT (8.89/0.71)

| | | | | | | | | dbsnp_siteG = DBSNP: ACCEPT (21.3/2.66)

| | | | | | | | | dbsnp_siteG = COSMIC: ACCEPT (0.0)

| | | | | | | | | dbsnp_siteG = DBSNP+COSMIC: DISCARD (0.63/0.0)

| | | | | | | | powerX > 0.099721: DISCARD (3.87/1.13)

| | | | | | | total_readsX > 35

| | | | | | | | judgementX = KEEP: ACCEPT (12.43/3.9)

| | | | | | | | judgementX = REJECT

| | | | | | | | | dbsnp_siteG = NOVEL

| | | | | | | | | | coveredG = COVERED: DISCARD (24.16/3.75)

| | | | | | | | | | coveredG = UNCOVERED

| | | | | | | | | | | t_del_countG <= 0: DISCARD (4.93/1.68)

| | | | | | | | | | | t_del_countG > 0: ACCEPT (2.33/0.03)

| | | | | | | | | dbsnp_siteG = DBSNP

| | | | | | | | | | snp_qualG <= 4.786: DISCARD (18.64/0.83)

| | | | | | | | | | snp_qualG > 4.786

| | | | | | | | | | | tumor_powerG <= 0.851984

| | | | | | | | | | | | n_ref_countG <= 4: DISCARD (7.01/0.0)

| | | | | | | | | | | | n_ref_countG > 4

| | | | | | | | | | | | | SB3G <= 0

| | | | | | | | | | | | | | tumor_powerG <= 0.260965: ACCEPT (5.43/1.99)

| | | | | | | | | | | | | | tumor_powerG > 0.260965: DISCARD (7.51/0.0)

| | | | | | | | | | | | | SB3G > 0

| | | | | | | | | | | | | | coveredX = COVERED: ACCEPT (32.27/9.63)

| | | | | | | | | | | | | | coveredX = UNCOVERED: DISCARD (3.19/0.67)

| | | | | | | | | | | tumor_powerG > 0.851984: DISCARD (10.9/0.44)

| | | | | | | | | dbsnp_siteG = COSMIC: DISCARD (7.77/3.0)

| | | | | | | | | dbsnp_siteG = DBSNP+COSMIC: DISCARD (16.06/0.57)

| | | | | t_del_countG > 5

| | | | | | total_readsG <= 47

| | | | | | | normal_fX <= 0.140845

| | | | | | | | t_alt_max_mapqG <= 14

| | | | | | | | | n_alt_sumG <= 33: DISCARD (4.42/1.0)

| | | | | | | | | n_alt_sumG > 33: ACCEPT (3.72/0.03)

| | | | | | | | t_alt_max_mapqG > 14

| | | | | | | | | snp_qualG <= 4.281: DISCARD (5.08/1.36)

| | | | | | | | | snp_qualG > 4.281: ACCEPT (74.57/5.75)

| | | | | | | normal_fX > 0.140845

| | | | | | | | SB3X <= 1: ACCEPT (6.27/1.96)

| | | | | | | | SB3X > 1: DISCARD (10.01/2.16)

| | | | | | total_readsG > 47

| | | | | | | t_alt_countG <= 6

| | | | | | | | refG <= 32

| | | | | | | | | altG <= 5

| | | | | | | | | | n_q20_countG <= 30

| | | | | | | | | | | SB1G <= 17: DISCARD (19.16/1.02)

| | | | | | | | | | | SB1G > 17: ACCEPT (2.27/0.26)

| | | | | | | | | | n_q20_countG > 30: ACCEPT (2.27/0.02)

| | | | | | | | | altG > 5: ACCEPT (8.34/1.25)

| | | | | | | | refG > 32: DISCARD (21.64/0.24)

| | | | | | | t_alt_countG > 6: ACCEPT (3.03/0.03)

| | | | snp_qualG > 11.373

| | | | | init_n_lodG <= -5.406265

| | | | | | map_Q0_readsG <= 3

| | | | | | | t_ref_countX <= 23

| | | | | | | | normal_fX <= 0.135593: ACCEPT (30.14/4.11)

| | | | | | | | normal_fX > 0.135593

| | | | | | | | | dbsnp_siteG = NOVEL

| | | | | | | | | | t_ins_countG <= 1: ACCEPT (8.28/1.59)

| | | | | | | | | | t_ins_countG > 1: DISCARD (3.03/0.0)

| | | | | | | | | dbsnp_siteG = DBSNP

| | | | | | | | | | altG <= 7: ACCEPT (4.79/0.37)

| | | | | | | | | | altG > 7

| | | | | | | | | | | t_del_countG <= 4

| | | | | | | | | | | | SB2G <= 15: DISCARD (15.22/0.01)

| | | | | | | | | | | | SB2G > 15

| | | | | | | | | | | | | map_Q0_readsG <= 0: ACCEPT (3.88/0.88)

| | | | | | | | | | | | | map_Q0_readsG > 0: DISCARD (4.43/1.0)

| | | | | | | | | | | t_del_countG > 4: ACCEPT (4.26/1.26)

| | | | | | | | | dbsnp_siteG = COSMIC: DISCARD (0.0)

| | | | | | | | | dbsnp_siteG = DBSNP+COSMIC: DISCARD (2.44/0.0)

| | | | | | | t_ref_countX > 23

| | | | | | | | n_ref_countG <= 34: DISCARD (139.34/22.09)

| | | | | | | | n_ref_countG > 34: ACCEPT (7.16/1.5)

| | | | | | map_Q0_readsG > 3

| | | | | | | tot_depthG <= 21: DISCARD (14.92/2.29)

| | | | | | | tot_depthG > 21

| | | | | | | | powerX <= 0.999968: ACCEPT (51.5/3.81)

| | | | | | | | powerX > 0.999968

| | | | | | | | | snp_qualG <= 15.012: DISCARD (6.81/0.17)

| | | | | | | | | snp_qualG > 15.012

| | | | | | | | | | normal_fG <= 0.204082: ACCEPT (18.62/3.95)

| | | | | | | | | | normal_fG > 0.204082: DISCARD (4.06/1.0)

| | | | | init_n_lodG > -5.406265

| | | | | | t_ref_max_mapqG <= 41

| | | | | | | normal_power_nspG <= 0.992708: DISCARD (17.05/0.89)

| | | | | | | normal_power_nspG > 0.992708: ACCEPT (10.4/4.19)

| | | | | | t_ref_max_mapqG > 41

| | | | | | | n_q20_countG <= 40

| | | | | | | | normal_fX <= 0.226087

| | | | | | | | | init_t_lodX <= -5.057324

| | | | | | | | | | t_ins_countX <= 2

| | | | | | | | | | | normal_powerX <= 0.999806: DISCARD (14.11/4.94)

| | | | | | | | | | | normal_powerX > 0.999806: ACCEPT (99.24/22.01)

| | | | | | | | | | t_ins_countX > 2: DISCARD (5.79/0.38)

| | | | | | | | | init_t_lodX > -5.057324: ACCEPT (366.26/34.86)

| | | | | | | | normal_fX > 0.226087

| | | | | | | | | coveredX = COVERED

| | | | | | | | | | coveredG = COVERED

| | | | | | | | | | | snp_filterG <= 0: ACCEPT (37.14/15.56)

| | | | | | | | | | | snp_filterG > 0: DISCARD (2.28/0.03)

| | | | | | | | | | coveredG = UNCOVERED

| | | | | | | | | | | t_alt_max_mapqX <= 43: ACCEPT (5.28/0.68)

| | | | | | | | | | | t_alt_max_mapqX > 43: DISCARD (12.37/0.84)

| | | | | | | | | coveredX = UNCOVERED: ACCEPT (16.57/4.6)

| | | | | | | n_q20_countG > 40

| | | | | | | | tumor_fG <= 0.248366

| | | | | | | | | normal_best_gtG = AA

| | | | | | | | | | init_n_lodG <= 13.105931: ACCEPT (11.0/1.12)

| | | | | | | | | | init_n_lodG > 13.105931: DISCARD (3.48/0.0)

| | | | | | | | | normal_best_gtG = GG

| | | | | | | | | | snp_qualG <= 17.671: DISCARD (4.72/0.14)

| | | | | | | | | | snp_qualG > 17.671: ACCEPT (10.66/2.35)

| | | | | | | | | normal_best_gtG = CC

| | | | | | | | | | SB4G <= 0: DISCARD (9.55/0.01)

| | | | | | | | | | SB4G > 0

| | | | | | | | | | | t_lod_fstar_forG <= 5.974369: ACCEPT (4.05/0.04)

| | | | | | | | | | | t_lod_fstar_forG > 5.974369: DISCARD (2.02/0.0)

| | | | | | | | | normal_best_gtG = TT

| | | | | | | | | | dbsnp_siteG = NOVEL: DISCARD (2.79/0.0)

| | | | | | | | | | dbsnp_siteG = DBSNP

| | | | | | | | | | | t_alt_max_mapqG <= 45: ACCEPT (5.5/0.06)

| | | | | | | | | | | t_alt_max_mapqG > 45: DISCARD (2.02/0.0)

| | | | | | | | | | dbsnp_siteG = COSMIC: ACCEPT (0.0)

| | | | | | | | | | dbsnp_siteG = DBSNP+COSMIC: ACCEPT (1.01/0.01)

| | | | | | | | | normal_best_gtG = AG

| | | | | | | | | | normal_fG <= 0.065934: ACCEPT (2.02/0.02)

| | | | | | | | | | normal_fG > 0.065934: DISCARD (4.94/0.0)

| | | | | | | | | normal_best_gtG = GT: DISCARD (0.0)

| | | | | | | | | normal_best_gtG = CT: ACCEPT (3.48/0.48)

| | | | | | | | | normal_best_gtG = AT: DISCARD (1.46/0.0)

| | | | | | | | | normal_best_gtG = AC: DISCARD (4.05/0.0)

| | | | | | | | | normal_best_gtG = CG: DISCARD (2.02/0.0)

| | | | | | | | tumor_fG > 0.248366: ACCEPT (15.05/0.6)

| | | normal_fG > 0.218014

| | | | normal_fX <= 0.28

| | | | | t_q20_countX <= 17

| | | | | | tumor_powerG <= 0.58293: DISCARD (12.45/1.05)

| | | | | | tumor_powerG > 0.58293: ACCEPT (22.67/4.49)

| | | | | t_q20_countX > 17

| | | | | | n_ref_countG <= 16: DISCARD (209.28/18.62)

| | | | | | n_ref_countG > 16

| | | | | | | normal_power_nspX <= 0.999931: ACCEPT (5.82/0.76)

| | | | | | | normal_power_nspX > 0.999931: DISCARD (35.35/6.33)

| | | | normal_fX > 0.28: DISCARD (465.49/7.26)

| | total_readsG > 1434

| | | SB4G <= 5: DISCARD (1054.32/5.69)

| | | SB4G > 5

| | | | AT_FmadG <= 7.5: DISCARD (22.24/0.01)

| | | | AT_FmadG > 7.5: ACCEPT (4.04/0.04)

| n_alt_sumG > 202

| | normal_fG <= 0.287356

| | | total_readsG <= 1744

| | | | snp_qualG <= 74.505

| | | | | normal_fG <= 0.214431

| | | | | | snp_qualG <= 12.162

| | | | | | | coveredX = COVERED: DISCARD (284.27/3.85)

| | | | | | | coveredX = UNCOVERED

| | | | | | | | SB3G <= 86: DISCARD (12.41/1.5)

| | | | | | | | SB3G > 86: ACCEPT (3.09/0.08)

| | | | | | snp_qualG > 12.162

| | | | | | | tumor_fG <= 0.231579

| | | | | | | | SB2X <= 97

| | | | | | | | | judgementX = KEEP: ACCEPT (3.48/0.31)

| | | | | | | | | judgementX = REJECT

| | | | | | | | | | normal_fX <= 0.26

| | | | | | | | | | | map_Q0_readsG <= 118

| | | | | | | | | | | | t_alt_countG <= 16

| | | | | | | | | | | | | snp_qualG <= 36.873

| | | | | | | | | | | | | | normal_best_gtX = TT: DISCARD (4.74/0.06)

| | | | | | | | | | | | | | normal_best_gtX = AA: DISCARD (2.67/0.02)

| | | | | | | | | | | | | | normal_best_gtX = GG

| | | | | | | | | | | | | | | t_lod_fstar_revX <= 4.93973: DISCARD (9.0/0.09)

| | | | | | | | | | | | | | | t_lod_fstar_revX > 4.93973: ACCEPT (2.21/0.18)

| | | | | | | | | | | | | | normal_best_gtX = CC: DISCARD (10.15/1.1)

| | | | | | | | | | | | | | normal_best_gtX = CT

| | | | | | | | | | | | | | | n_q20_countG <= 42: DISCARD (22.77/1.17)

| | | | | | | | | | | | | | | n_q20_countG > 42

| | | | | | | | | | | | | | | | init_t_lodX <= -4.544543

| | | | | | | | | | | | | | | | | total_readsG <= 119: ACCEPT (2.5/0.07)

| | | | | | | | | | | | | | | | | total_readsG > 119: DISCARD (11.84/0.13)

| | | | | | | | | | | | | | | | init_t_lodX > -4.544543: ACCEPT (22.8/8.17)

| | | | | | | | | | | | | | normal_best_gtX = AG

| | | | | | | | | | | | | | | SB3G <= 3

| | | | | | | | | | | | | | | | tumor_fX <= 0.126984

| | | | | | | | | | | | | | | | | coveredX = COVERED

| | | | | | | | | | | | | | | | | | altG <= 11: DISCARD (9.32)

| | | | | | | | | | | | | | | | | | altG > 11

| | | | | | | | | | | | | | | | | | | snp_qualX <= 30.941: DISCARD (10.47/1.78)

| | | | | | | | | | | | | | | | | | | snp_qualX > 30.941: ACCEPT (5.55/1.14)

| | | | | | | | | | | | | | | | | coveredX = UNCOVERED: ACCEPT (4.35/1.32)

| | | | | | | | | | | | | | | | tumor_fX > 0.126984: ACCEPT (4.43/0.35)

| | | | | | | | | | | | | | | SB3G > 3: DISCARD (11.0/0.15)

| | | | | | | | | | | | | | normal_best_gtX = CG

| | | | | | | | | | | | | | | tumor_powerG <= 0.9982: DISCARD (10.9/0.07)

| | | | | | | | | | | | | | | tumor_powerG > 0.9982

| | | | | | | | | | | | | | | | dbsnp_siteX = NOVEL: ACCEPT (2.21/0.18)

| | | | | | | | | | | | | | | | dbsnp_siteX = DBSNP

| | | | | | | | | | | | | | | | | init_n_lodG <= -14.65134: DISCARD (6.72/1.11)

| | | | | | | | | | | | | | | | | init_n_lodG > -14.65134: ACCEPT (3.2/0.2)

| | | | | | | | | | | | | | | | dbsnp_siteX = COSMIC: ACCEPT (0.0)

| | | | | | | | | | | | | | | | dbsnp_siteX = DBSNP+COSMIC: ACCEPT (0.0)

| | | | | | | | | | | | | | normal_best_gtX = AT

| | | | | | | | | | | | | | | contaminant_lodG <= -0.420604: ACCEPT (2.01/0.01)

| | | | | | | | | | | | | | | contaminant_lodG > -0.420604: DISCARD (8.95/0.1)

| | | | | | | | | | | | | | normal_best_gtX = GT

| | | | | | | | | | | | | | | map_Q0_readsX <= 7: DISCARD (7.67/2.07)

| | | | | | | | | | | | | | | map_Q0_readsX > 7: ACCEPT (2.67/0.21)

| | | | | | | | | | | | | | normal_best_gtX = AC

| | | | | | | | | | | | | | | normal_power_wspG <= 0.999912: ACCEPT (2.3/0.25)

| | | | | | | | | | | | | | | normal_power_wspG > 0.999912: DISCARD (16.6/3.11)

| | | | | | | | | | | | | snp_qualG > 36.873

| | | | | | | | | | | | | | coveredX = COVERED

| | | | | | | | | | | | | | | dbsnp_siteG = NOVEL

| | | | | | | | | | | | | | | | normal_best_gtG = AA: ACCEPT (0.0)

| | | | | | | | | | | | | | | | normal_best_gtG = GG: ACCEPT (0.0)

| | | | | | | | | | | | | | | | normal_best_gtG = CC: ACCEPT (0.0)

| | | | | | | | | | | | | | | | normal_best_gtG = TT: ACCEPT (0.0)

| | | | | | | | | | | | | | | | normal_best_gtG = AG

| | | | | | | | | | | | | | | | | t_lod_fstar_revG <= 3.614914: ACCEPT (4.68/0.2)

| | | | | | | | | | | | | | | | | t_lod_fstar_revG > 3.614914: DISCARD (2.18/0.0)

| | | | | | | | | | | | | | | | normal_best_gtG = GT: DISCARD (3.07/0.15)

| | | | | | | | | | | | | | | | normal_best_gtG = CT

| | | | | | | | | | | | | | | | | tot_depthG <= 142: ACCEPT (5.49/1.29)

| | | | | | | | | | | | | | | | | tot_depthG > 142: DISCARD (3.3/0.12)

| | | | | | | | | | | | | | | | normal_best_gtG = AT: ACCEPT (5.0/1.52)

| | | | | | | | | | | | | | | | normal_best_gtG = AC: ACCEPT (1.17/0.17)

| | | | | | | | | | | | | | | | normal_best_gtG = CG: ACCEPT (1.49/0.49)

| | | | | | | | | | | | | | | dbsnp_siteG = DBSNP

| | | | | | | | | | | | | | | | t_ref_max_mapqG <= 41: DISCARD (2.02/0.0)

| | | | | | | | | | | | | | | | t_ref_max_mapqG > 41: ACCEPT (29.11/6.63)

| | | | | | | | | | | | | | | dbsnp_siteG = COSMIC: ACCEPT (0.0)

| | | | | | | | | | | | | | | dbsnp_siteG = DBSNP+COSMIC: DISCARD (1.01/0.0)

| | | | | | | | | | | | | | coveredX = UNCOVERED

| | | | | | | | | | | | | | | p_allX <= -1.199: DISCARD (8.33/0.21)

| | | | | | | | | | | | | | | p_allX > -1.199: ACCEPT (4.18/1.07)

| | | | | | | | | | | | t_alt_countG > 16

| | | | | | | | | | | | | SB4X <= 4: DISCARD (38.72/1.75)

| | | | | | | | | | | | | SB4X > 4

| | | | | | | | | | | | | | n_alt_countG <= 11: ACCEPT (5.01/1.01)

| | | | | | | | | | | | | | n_alt_countG > 11

| | | | | | | | | | | | | | | SB2X <= 28: ACCEPT (4.08/1.4)

| | | | | | | | | | | | | | | SB2X > 28: DISCARD (14.76/0.34)

| | | | | | | | | | | map_Q0_readsG > 118: DISCARD (30.89/1.18)

| | | | | | | | | | normal_fX > 0.26

| | | | | | | | | | | map_Q0_readsX <= 3: DISCARD (55.71/0.7)

| | | | | | | | | | | map_Q0_readsX > 3

| | | | | | | | | | | | t_del_countX <= 22

| | | | | | | | | | | | | t_alt_max_mapqG <= 22

| | | | | | | | | | | | | | t_ref_max_mapqG <= 55: DISCARD (3.12)

| | | | | | | | | | | | | | t_ref_max_mapqG > 55

| | | | | | | | | | | | | | | dbsnp_siteG = NOVEL: DISCARD (2.57/0.14)

| | | | | | | | | | | | | | | dbsnp_siteG = DBSNP: ACCEPT (4.87/0.82)

| | | | | | | | | | | | | | | dbsnp_siteG = COSMIC: ACCEPT (0.0)

| | | | | | | | | | | | | | | dbsnp_siteG = DBSNP+COSMIC: ACCEPT (0.0)

| | | | | | | | | | | | | t_alt_max_mapqG > 22: DISCARD (18.85/0.19)

| | | | | | | | | | | | t_del_countX > 22: ACCEPT (2.22/0.2)

| | | | | | | | SB2X > 97: DISCARD (181.93/10.29)

| | | | | | | tumor_fG > 0.231579

| | | | | | | | normal_fX <= 0.184783

| | | | | | | | | n_alt_countG <= 12: ACCEPT (34.47/5.51)

| | | | | | | | | n_alt_countG > 12

| | | | | | | | | | Power_Pos_SBG <= 0.999993: DISCARD (10.55/1.01)

| | | | | | | | | | Power_Pos_SBG > 0.999993

| | | | | | | | | | | AT_FmadG <= 11.5: DISCARD (5.57/0.01)

| | | | | | | | | | | AT_FmadG > 11.5: ACCEPT (7.08/1.07)

| | | | | | | | normal_fX > 0.184783

| | | | | | | | | tumor_fX <= 0.303571: DISCARD (22.65/1.09)

| | | | | | | | | tumor_fX > 0.303571

| | | | | | | | | | normal_best_gtX = TT: DISCARD (0.0)

| | | | | | | | | | normal_best_gtX = AA: DISCARD (0.0/0.0)

| | | | | | | | | | normal_best_gtX = GG: DISCARD (0.02/0.0)

| | | | | | | | | | normal_best_gtX = CC: DISCARD (0.03/0.0)

| | | | | | | | | | normal_best_gtX = CT: DISCARD (7.98/1.38)

| | | | | | | | | | normal_best_gtX = AG

| | | | | | | | | | | n_ref_countG <= 55: ACCEPT (4.45/0.02)

| | | | | | | | | | | n_ref_countG > 55: DISCARD (4.47/0.0)

| | | | | | | | | | normal_best_gtX = CG: ACCEPT (1.11/0.06)

| | | | | | | | | | normal_best_gtX = AT: DISCARD (1.28/0.06)

| | | | | | | | | | normal_best_gtX = GT: DISCARD (3.51/0.75)

| | | | | | | | | | normal_best_gtX = AC: ACCEPT (1.12/0.06)

| | | | | normal_fG > 0.214431: DISCARD (795.6/36.35)

| | | | snp_qualG > 74.505

| | | | | normal_fG <= 0.205732

| | | | | | t_del_countG <= 72

| | | | | | | tumor_powerG <= 0.999407

| | | | | | | | normal_fX <= 0.137931: ACCEPT (4.3/0.91)

| | | | | | | | normal_fX > 0.137931: DISCARD (8.27/0.19)

| | | | | | | tumor_powerG > 0.999407: ACCEPT (223.66/29.41)

| | | | | | t_del_countG > 72

| | | | | | | n_alt_countG <= 7: ACCEPT (2.02/0.02)

| | | | | | | n_alt_countG > 7: DISCARD (13.29/0.01)

| | | | | normal_fG > 0.205732

| | | | | | n_alt_sumX <= 97: ACCEPT (9.91/1.72)

| | | | | | n_alt_sumX > 97

| | | | | | | t_ref_max_mapqG <= 57

| | | | | | | | normal_fX <= 0.260465: ACCEPT (3.04/0.04)

| | | | | | | | normal_fX > 0.260465: DISCARD (5.79)

| | | | | | | t_ref_max_mapqG > 57: DISCARD (156.06/14.09)

| | | total_readsG > 1744

| | | | t_alt_countG <= 152: DISCARD (3569.33/35.33)

| | | | t_alt_countG > 152

| | | | | n_ref_sumG <= 29056: DISCARD (47.51/1.03)

| | | | | n_ref_sumG > 29056: ACCEPT (6.07/1.06)

| | normal_fG > 0.287356

| | | n_alt_sumX <= 67

| | | | judgementX = KEEP: ACCEPT (7.05/2.32)

| | | | judgementX = REJECT

| | | | | map_Q0_readsX <= 1

| | | | | | tumor_powerX <= 0.292001: ACCEPT (16.74/5.62)

| | | | | | tumor_powerX > 0.292001: DISCARD (36.76/4.26)

| | | | | map_Q0_readsX > 1: DISCARD (67.27/2.04)

| | | n_alt_sumX > 67: DISCARD (17906.84/66.65)
